# Smart lasers tame complex spatiotemporal cavity dynamics

**DOI:** 10.1038/s41377-020-00426-0

**Published:** 2020-11-19

**Authors:** Philippe Grelu

**Affiliations:** Laboratoire ICB UMR 6303 CNRS, Université Bourgogne – Franche-Comté, 21000 Dijon, France

**Keywords:** Fibre lasers, Photonic devices

## Abstract

By associating multimode fibers, optical wavefront manipulation, and a feedback loop controlled by a genetic algorithm, researchers have demonstrated that nonlinear spatiotemporal dynamics can be flexed within the laser cavity to achieve a user-specified objective, such as the lasing wavelength, output power, beam profile or pulsed operation.

Laser technology has made tremendous progress over the past few decades, with widespread applications in all the major sectors of human activity. However, the ideal ready-to-use laser system is mainly thought to be a box with an on/off switch enabling fire of a factory-fixed laser output. This concept is certainly convenient for the majority of laser application processes requiring stable laser operation with well-defined specifications concerning its spectral, temporal, and spatial features. Nevertheless, there is a huge avenue of research to enhance the number of laser output parameters that can be adjusted by the end user in a way similar to that in electronics, namely, function generators offering a wide range of output options. To generate nearly arbitrary ultrafast optical waveforms, the shaping of laser output pulses in the Fourier spectral domain has proved to be an invaluable technique^1^. In the spectral domain, there are also important efforts to develop integrated optical frequency synthesizers combining tunability with high purity of the generated frequency^2^.

However, instead of manipulating the optical field outside the laser cavity, which implies substantial power losses, it could be advantageous to tailor the laser field within the optical cavity. Laser physicists know that in the presence of numerous cavity modes, gain competition, and fluctuations from spontaneous emission, the laser dynamics supports surprising morphing capacities to adapt to the constraints imposed, evolving toward minimal roundtrip losses. This ability operates in both the spatial domain (transverse modes), which corresponds to optical morphogenesis^3^, and in the temporal domain (longitudinal modes), with laser mode locking as the most vivid illustration. Therefore, the recent spatiotemporal mode-locking of lasers having multiple longitudinal as well as transverse modes^4^, while representing a technical challenge in terms of a suitable selection of cavity parameters, appears retrospectively as a brilliant expression of the self-organization capacities of a dissipative nonlinear laser system with extended dimensionality.

Fiber lasers offer great flexibility in cavity design, allowing the combination of various integrated components with reduced coupling losses in a cost-effective way. They are also prone to an increase in the number of parameters affecting the laser dynamics. Some cavity parameters may not be known precisely, such as stress-induced birefringence and mode coupling parameters, which furthermore distribute unevenly along the cavity, following the specific arrangement of fibers and associated components. Therefore, numerical simulations, while offering appreciable guidelines, are often of little help to operate a fiber laser setup presenting great versatility in its output. In laboratory experiments, this typically implies trial-and-error manual adjustment for an appreciable lapse of time before reaching a desired regime of laser emission.

To address the complex task of reaching an optimized laser output from the actuation of a set of interfaced cavity parameters, the use of genetic algorithms was proposed a few years ago^5^. Genetic and evolutionary algorithms are well suited to the optimization problem of complex functions having multiple local extrema. Notably, they have been used to pilot optical pulse shaping—in the time domain—to optimize the coherent control of chemical reactions^6^, or to reach efficient focusing through turbid media—in the spatial domain^7^. However, the intracavity control of laser dynamics via genetic algorithms raises an additional complexity. The initial investigation, which targeted self-starting mode locking, highlighted the challenge of optimizing the laser regime in the presence of hysteresis and bifurcation dynamics^5^. Important experimental developments followed, involving, for instance, the conjunction of several characterization devices to define a compound merit function, approaching the appreciation of a trained experimentalist about the mode-locking performance^8^. Recently, the implementation of real-time spectral characterization methods significantly improved the characterization process by reducing the latency in the scoring of the numerous laser regimes obtained throughout the optimization process, as well as by simplifying the required instrumentation^9–11^.

In a recent study^12^, Wei et al. succeeded in combining the spatiotemporal complexity of a laser cavity, comprising multimode optical fibers, with genetic algorithm optimization. To effectively manipulate this large number of laser cavity modes, they introduced an important set of actuated parameters by incorporating a spatial light modulator (SLM) within the laser cavity. In this way, the intracavity wavefront shaping has been controlled by a genetic algorithm to optimize a user-defined merit function. The schematic association of a multimode fiber laser with the SLM feedback controlled by a genetic algorithm is displayed in Fig. 1. Reflecting the vast possibilities of spatiotemporal laser dynamics, the authors successively requested a genetic algorithm to select a desired laser wavelength, to optimize the output power, to improve the beam profile, and to trigger pulsed operation entailing spatiotemporal mode locking. These various optimizations were conducted through the sole manipulation of the spatial beam profile inside the cavity. This quite amazing trait reflects the strong coupling between space and time field variables in a spatiotemporal laser. Noting that these optimizations have been performed independently, the report naturally raises the question of the feasibility of multicriteria optimizations as extensions of the tests undertaken previously in the case of purely temporal fiber lasers^8–10^. For instance, one could envisage selecting a specific pulsed regime with a given mode profile composition emitting around a desired central wavelength. This would probably require the incorporation of additional controllable cavity parameters, implying a larger testing set and, likely, an increased number of iterations for the genetic algorithm. Therefore, important fundamental and practical questions remain, such as those regarding the sets of laser dynamics that can be accessed and suitably characterized, reached through optimization and routinely replicated. Obviously, the characterization of lasers with spatiotemporal dynamics requires new technical upgrades, for instance, implementing hyperspectral imaging. These advanced spatiotemporal characterization methods could challenge the data processing capacity to keep up an acceptable optimization time that should remain comparable to the warm-up time of most instrumentation. Therefore, the recent publication from Wei et al.^12^ will trigger subsequent investigations from the scientific community at the interface of machine learning, laser dynamics, and advanced optical characterization, with possible breakthroughs in the development of “smart lasers” for applicative needs.

**Fig. 1:**
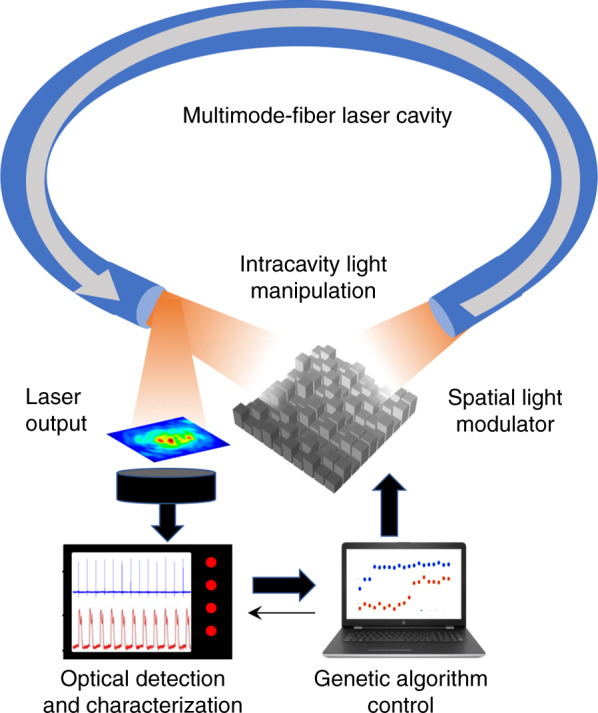
Schematic of a smart spatiotemporal fiber laser. Within the laser cavity, the optical field wavefront is manipulated in accordance with the process of a genetic algorithm, which optimizes a merit function that is defined by the user and is assessed by consecutive measurements of the laser output
